# 

**Published:** 1894-10-13

**Authors:** 


"Johannis" THE KING OF NATURAL TABLE WATERS. "Johannis"

### THE HOSPITAL

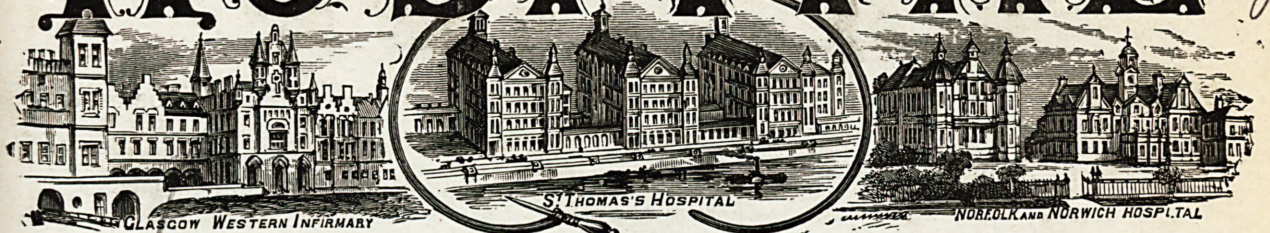

No. 420. Vol. XVII.

Saturday, Oct. 13th, 1894.

**WITH EXTRA NURSING SUPPLEMENT.**
[Registered at the General Post Office as a Newspaper for transmission in the United Kingdom and Abroad.]

**PRICE TWOPENCE.**

Per Annum, 10s. 6d., post free.

Monthly Parts, 6d.; post free, 8d.

Ditto per Annum, 8s. 6d., post free.

